# Treatment of sarcoidosis-an opinion

**DOI:** 10.36416/1806-3756/e20250330

**Published:** 2025-11-19

**Authors:** Eduardo Pamplona Bethlem, Marcos de Carvalho Bethlem, Paolo Spa gnolo

**Affiliations:** 1. Disciplina de Pneumologia, Faculdade de Medicina, Universidade Federal do Estado do Rio de Janeiro - UNIRIO - Rio de Janeiro (RJ) Brasil.; 2. Laboratório de Endoscopia Respiratória, Universidade Federal do Rio de Janeiro - UFRJ - Rio de Janeiro (RJ) Brasil.; 3. Hospital São Lucas, Rio de Janeiro (RJ) Brasil.; 4. Hospital Glória D’Or, Rio de Janeiro (RJ) Brasil.; 5. Respiratory Medicine Department and Respiratory Medicine Residency Program, University of Padua, Padua, Italy.

Sarcoidosis is a systemic granulomatous disease of unknown etiology, being characterized by the formation of noncaseating granulomas in multiple organs. The clinical presentation of sarcoidosis is highly variable, and in most cases the disease follows a benign course, with high rates of spontaneous resolution. This variability has hindered the development of robust clinical trials to define optimal therapeutic strategies, leading to treatment decisions that are largely based on expert opinion.^1^

In certain subsets of patients, particularly those who are asymptomatic or have minimal organ involvement, a “watchful waiting” approach without immediate treatment may be appropriate. Conversely, chronic and insidious forms of sarcoidosis tend to follow a protracted course and may carry a worse prognosis, necessitating therapeutic intervention.^1^ ^,^ ^2^

First-line treatment typically involves corticosteroids. However, because of the potential for significant side effects with long-term corticosteroid use, corticosteroid-sparing agents such as methotrexate are considered for use as second-line therapies.^1^ Corticosteroids exert most of their clinical effects within the initial months of treatment, with limited additional benefit thereafter and a progressively higher risk of adverse effects. Methotrexate, on the other hand, reaches its maximal therapeutic effect only after several months of use and is associated with fewer side effects. This pharmacological profile raises the question of whether these characteristics could be leveraged to optimize therapeutic response. 

Recent data from a randomized trial of methotrexate vs. prednisone in pulmonary sarcoidosis demonstrated the noninferiority of methotrexate when compared with corticosteroids as a first-line therapy in pulmonary sarcoidosis.^2^ This supports an “off-label” approach that one of us (EPB) has employed for some time now, i.e., using a combination of corticosteroids and methotrexate from the outset in cases of sarcoidosis with signs of severity and/or chronicity. When corticosteroids and methotrexate are initiated simultaneously, the former provide a rapid therapeutic effect in the early phase, whereas the latter begins to exert its efficacy at approximately three months after treatment initiation. By this time, corticosteroids are often associated with more adverse effects than benefits. Therefore, starting both treatments together allows for corticosteroid tapering and discontinuation at approximately three months after treatment initiation without triggering disease relapse, given that methotrexate has reached its full therapeutic potential by then. This combined approach in comparison with conventional monotherapy is an interesting point to be studied. Preliminary impressions suggest a lower incidence of side effects and a similarly effective therapeutic response.^3^

In conclusion, although corticosteroids remain the cornerstone of sarcoidosis treatment, the use of methotrexate as a first-line agent, either alone or in combination, offers a promising strategy to optimize therapeutic outcomes and minimize adverse effects. 
